# Accounting for Timing when Assessing Health-Related Policies

**DOI:** 10.1017/bca.2018.29

**Published:** 2019-01-26

**Authors:** Karl Claxton, Miqdad Asaria, Collins Chansa, Julian Jamison, James Lomas, Jessica Ochalek, Mike Paulden

**Affiliations:** 1 **Miqdad Asaria:** The London School of Economics and Political Science (LSE), Amrita Institute of Medical Sciences and Research Centre, London WC2A 2AE, UK, e-mail: m.asaria@lse.ac.uk; 2 **Collins Chansa:** World Bank Group, Lusaka 10101, Zambia, e-mail: cchansa@worldbank.org; 3 **Julian Jamison:** University of Exeter Business School, Exeter EX4 4PU, UK, e-mail: J.Jamison@exeter.ac.uk; 4 **James Lomas:** University of York, Centre for Health Economics, York YO10 5DD, UK, e-mail: james.lomas@york.ac.uk; 5 **Jessica Ochalek:** University of York, Centre for Health Economics York YO10 5DD, UK, e-mail: jessica.ochalek@york.ac.uk; 6 **Mike Paulden:** University of Alberta, School of Public Health Edmonton, Alberta T6G 1C9, Canada, e-mail: paulden@ualberta.ca

**Keywords:** Health, I1, O1, O2

## Abstract

The primary focus of this paper is to offer guidance on the analysis of time streams of effects that a project may have so that they can be discounted appropriately. This requires a framework that identifies the common parameters that need to be assessed, whether conducting cost-effectiveness or benefit-cost analysis. The quantification and conversion of the time streams of different effects into their equivalent health, health care cost or consumption effects avoids embedding multiple arguments in discounting policies. This helps to identify where parameters are likely to differ in particular contexts, what type of evidence would be relevant, what is currently known and how this evidence might be strengthened. The current evidence available to support the assessment of the key parameters is discussed and possible estimates and default assumptions are suggested. Reporting the results in an extensive way is recommended. This makes the assessments required explicit so the impact of alternative assumptions can be explored and analysis updated as better estimates evolve. Some projects will have effects across different countries where some or all of these parameters will differ. Therefore, the net present value of a project will be the sum of the country specific net present values rather than the sum of effects across countries discounted at some common rate.

## 1 Introduction

A decision to introduce a policy (e.g., public health, educational, environmental etc.) or implement a project (e.g., a health technology or programme of care for a particular indication) may offer some immediate health benefits for the current population but, in many circumstances, the health benefits will occur in future periods. Other projects are intended to reduce the risk of future events for the current population and/or reduce risks for future incident patients, so the health benefits they offer will not be fully realized for many years. Future benefits are not restricted to health but may also include impacts on private consumption, changes in future health care costs and other forms of public expenditure as well as social objectives of particular interest to the decision maker. Similarly, different policy choices and projects will not just impose health care and other costs in the current period but in future periods as well.

The question is how account should be taken of when health care and other costs are incurred and health and other benefits are received. The intention is to offer clarity about principles, the key parameters required and the evidence currently available to inform assessments of them, so that decision makers in low- and middle-income countries (LMICs) and other stakeholders, are better placed to judge what would be an appropriate analysis of the time streams of the effects that a project may have and the discount policy to apply to them in a particular context. This includes how global bodies, which make recommendations (e.g., World Health Organisation), purchase health technologies (e.g., Global Fund) or prioritize the development of new ones (e.g., Bill and Melinda Gates Foundation), should judge the value of projects which have effects in many different settings where appropriate discounting of costs and benefits are likely to differ.

The primary focus of this paper is to offer guidance on the appropriate analysis of time streams of the effects that a project may have (i.e., on health, health care costs and consumption) so that they can then be discounted appropriately. This requires a framework within which cost-effectiveness analysis (CEA) and benefit-cost analysis (BCA) can be understood and that identifies the common key parameters that need to be assessed. This helps to identify where parameters are likely to differ in particular contexts, what type of evidence would be relevant to inform their assessment, what is currently known that is relevant to low- and middle-income settings and how this evidence might be strengthened. This also makes the assessments and unavoidable judgements required explicit so the impact of alternative assumptions can be explored and analysis updated as better estimates evolve.

A common conceptual framework of how time streams of effects on health, health care costs and consumption may be considered is set out in Section 2. This identifies the key parameters that need to be assessed, whether conducting BCA or CEA. The evidence currently available that might support their assessment in LMIC settings is discussed in Section 3 and possible estimates and default assumptions are suggested. These are summarized in Section 4 before suggesting how analysis might be most usefully reported, especially when a project has effects across different contexts where parameters are likely to differ. Finally, some suggestions of priorities for further research are made.

## 2 Conceptual framework

A common conceptual framework within which CEA and BCA can be understood and key parameters identified is initially set out for a project that only has effects on time streams of health and health care costs. This is extended to consider effects beyond health and health care costs, where the often complex reality of multiple sectors is initially simplified into two (collective health care expenditure and private consumption) to illustrate principles. The common parameters that need to be assessed are identified and the distinction between BCA and CEA is discussed.

### 2.1 The objective of the project is to improve health

Decision-making bodies and institutions can be viewed as the agents of a principal (e.g., a socially legitimate process such as government) which allocates resources, devolves powers and gives responsibility to pursue specific, measurable and therefore narrowly defined objectives, e.g., to improve health. The values implied by the outcome of this process (e.g., government implicitly or explicitly determining collective expenditure on health care) can be regarded as a partial but revealed expression of some unknown social welfare function that may include many conflicting arguments, e.g., health equity, social solidarity among many others that are difficult to specify let alone quantify (Drummond et al., 2015). In these circumstances economic analysis cannot be used to make claims about social welfare or the optimality of the resources allocated to health care. Its role is more modest, claiming to inform accountable decision-making by revealing implied values and exposing the implications of current levels of health and other public expenditure. It is this role that economic analysis has tended to play in health policy and underpins much of the evaluation of health care projects and CEAs that have been conducted (Drummond et al., 2015; Coast et al., 2008).^1^

#### 2.1.1 Why discount health?

In this context the reason to discount future health effects cannot appeal to the type of welfare arguments that underpin social time preference for consumption, but instead to the opportunity costs of financing health care. The health care costs of a project could have been invested elsewhere in the economy or used to reduce public borrowing at a real rate of return, which would provide more health care resources in the future and generate greater health benefits. Health care transforms resources into health so from the perspective of a social planner trading health care resources over time is to trade health. Therefore, if health care costs are discounted to reflect the opportunity cost of financing health care, their health effects must also be discounted.^2^ For example, real yields on government bonds reflect the marginal cost of increasing health care expenditure available to government (Paulden & Claxton, 2012; Paulden et al., 2016). In this context, the broader question of the social opportunity costs of public expenditure including the macroeconomic choice of levels and mix of taxation and borrowing can be regarded as the responsibility of government rather than spending departments or national and supranational decision-making and advisory bodies.^3^

#### 2.1.2 Representing the effects of projects

Estimates of the additional health care costs (Δ*c_h_*) and additional health effects (Δ*h*) of a project whether measured as quality-adjusted life years (QALYs) gained or disability-adjusted life years (DALYs) averted are commonly reported as incremental cost-effectiveness ratios (ICERs).^4^. These provide a useful summary of how much additional resource is required to achieve a measured improvement in health (the additional cost per QALY gained or DALY averted). Whether the intervention will improve health outcomes overall requires a comparison with a “threshold” (*k_h_*) that reflects the likely health opportunity costs, i.e., the improvement in health that would have been possible if the additional resources required had, instead, been made available for other health care activities. A project will improve health overall if the additional health benefits exceed the health opportunity costs associated with the additional health care costs that must be found from existing commitments, or that require additional expenditure that could have been devoted to other health care activities (Δ*h* > Δ *c*_*h*_/*k*_*h*_).^5^

##### Time stream of net health effects

Most projects offer time streams of health effects (Δ*h*_*t*_) and health care costs (Δ*c*_*ht*_) illustrated in Table 1. The additional health care costs in each period can be reported as the health opportunity costs (Δ*c*_*ht*_/*k*_*ht*_, in column (5) of Table 1) by applying a “threshold” that reflects an assessment of the marginal productivity of health care expenditure relevant to that period (*k*_*ht*_). The time streams of health benefits and health opportunity losses can then be discounted at a rate which reflects a social time preference for health (*r*_*h*_).

**Table 1:** Reporting the effects of a project with health benefits and health care costs.

	Effects of the project		Health effects		Equivalent health care resources		Equivalent consumption effects	
(1) Time	(2) Additional health benefits	(3) Additional health care costs	(4) Health gained	(5) Health loss	(6) Benefits	(7) Costs	(8) Benefits	(9) Costs
1	Δ*h*_1_	Δ*c_h_*_1_	Δ*_h_*_1_	Δ*_ch_*_1_/*k*_*h*__1_	*k*_*h*__1_ · Δ*h*_1_	Δ*c*_*h*__1_	*V*_*h*__1_ · Δ_*h*__1_	*V*_*h*__1_(Δ*c*_*h*__1_/*k*_*h*__1_)
—	—	—	—	—	—	—	—	—
*t*	Δ*ht*	Δ*c*_*ht*_	Δ*ht*	Δ*c*_*ht*_/*k*_*ht*_	*k*_*ht*_ · Δ*ht*	Δ*c*_*ht*_	*V*_*ht*_ · Δ*ht*	Δ_*ht*_(Δ*c*_*ht*_/*k*_*ht*_)
—	—	—	—	—	—	—	—	—
*T*	Δ_*hT*_	Δ*c*_*hT*_	Δ*h*_*T*_	Δ*c*_*hT*_/*k*_*hT*_	*k*_*hT*_ · Δ*h*_*T*_	Δ*c*_*hT*_	*V*_*hT*_ · Δ*h*_*T*_	*V*_*hT*_(Δ*c*_*hT*_/*k*_*hT*_)

The social choice of how much resource to devote to health care over time and the resulting health in each period reveals something about society’s willingness to trade current and future health. For example, the choice of the principal in setting the level of health expenditure in each period, based on expectations about how the marginal productivity of health care expenditure is likely to evolve, implies values for *k*_*ht*_. Therefore, a revealed social time preference for health^6^ can be based on the rate at which the principal can borrow or save (*r*_*s*_) and whether the “threshold” is expected to grow (*gk*_*h*_) because this indicates the relative value (in terms of health care resources) of current compared to future health (*r*_*h*_ = *r*_*s*_ – *gk*_*h*_) (Paulden & Claxton, 2012; Paulden et al., 2016).

##### Time stream of equivalent health care resources

Rather than represent the additional health care costs of the project as health opportunity losses, the health benefits can be valued as the additional health care resources which would have been required to deliver similar health benefits in that period by applying the relevant “threshold” to the health benefits (*k*_*ht*_ · Δ*h*_*t*_ in column 7). The time streams of equivalent health care resource benefits and costs can then be discounted at a rate which reflects the opportunity cost, faced by the principal, of increasing public health care expenditure, *r*_*s*_, (e.g., real yields on government bonds).

##### Reporting cost-effectiveness ratios

Most cost-effectiveness analyses of health care projects report ICERs rather than net health benefits (column 4–5) or the equivalent net effect on health care resources (column 6–7) (Phelps & Mushlin, 1991; Stinnett & Mullahy, 1998). An ICER (Δ*c*_*h*_/Δ*h*) must be compared to a single “threshold” relevant to the current period (*k*_*h*__1_). However, some account must be taken of expected changes in health opportunity costs. For example, if the cost-effectiveness “threshold” is expected to grow in real terms (*gk*_*h*_> 0), because the marginal productivity of health care expenditure is expected to decline (e.g., due to real growth in health expenditure), then future costs are less important because they will displace less health, or additional resources could deliver less health (Paulden et al., 2017). Therefore, reporting ICERs requires discounting the additional health care costs at a rate that accounts for any growth in the “threshold”, to reflect the relative importance of future costs (*r*_*h*_ + *gk*_*h*_).^7^ This differential or dual discounting reflects expected changes in the marginal productivity of health care expenditure as well as time preference for health (Claxton et al., 2011).

The widespread reporting of ICERs in CEA may reflect reluctance on the part of decision-making and advisory bodies to be explicit about how much health care systems can afford to pay to improve health and how this is likely to evolve over time. Until recently there has also been a lack of evidence about the likely health opportunity costs (Culyer et al., 2007). As a consequence implicit assessments have been embedded in how costs and health effects are discounted. This has contributed to a lack of clarity about discounting policy, what a cost-effectiveness “threshold” ought to represent and how it might be informed with evidence.

One key recommendation is that this and other forms of dual discounting should be avoided (see Section 4.2). Although ICERs might be a familiar and useful summary, the primary analysis should report time streams of health benefits and health care costs (columns 2 and 3 in Table 1), and their transformation into streams of health effects (columns 4 and 5 in Table 1) and streams of equivalent health care resources (columns 6 and 7 in Table 1) based on an explicit assessment of health opportunity costs.

##### Time stream of equivalent consumption effects

The health effects of the project in columns (4) and (5) of Table 1 can also be expressed as the equivalent consumption value of the health benefits (*V*_*ht*_ · Δ*h*_*t*_, column 8) and the heath opportunity losses (*V*_*ht*_(Δ*c*_*ht*_/*k*_*ht*_, column 9)) in each time period. This requires some assessment of the consumption value of health (*V*_*ht*_) and how it is likely to evolve over time. However, in this example, where there are only effects on health and health care costs, or where the social planner has decided that other effects should be set aside when considering this type of health care project,^8^ *V*_*ht*_ does not influence the decision because it simply rescales any net health benefit or net health loss (both sides of Δ*h* > Δ*c*_*h*_/*k*_*h*_ are multiplied by *V*_*ht*_). Since *k*_*ht*_ and *V*_*ht*_ cannot be assumed to be necessarily and always equal (see Section 3.4 for discussion of the reasoning and empirical evidence that suggests *k*_*ht*_ < *V*_*ht*_) health care costs cannot be treated as if they are private consumption costs and vice versa.

A “threshold” that reflects an assessment of health opportunity costs is necessary when comparing different health care projects competing for limited health care resources. However, it is also relevant when considering broader questions of whether public resources available for health care should be increased. For example, it helps to inform: (i) whether there may be a case for increasing health expenditure because *k*_*ht*_ < *V*_*ht*_ so some projects are rejected that would have offered net social benefits if total health expenditure was increased and (ii) how much of an increase in expenditure should be considered.^9^ It would be better to evaluate projects founded on an empirically based assessment of *k*_*ht*_ and *V*_*ht*_ and how they are expected to evolve, rather than assume that public finances will be immediately set to accommodate the project being evaluated.

#### 2.1.3 Nonhealth impacts and nonhealth care costs

Projects often impose costs or offer benefits beyond measures of health and health care expenditure. For example, there may be out of pocket costs and/or net production effects of improved survival and quality of life (e.g., Meltzer, 2013) as well as impacts on other social objectives. Other types of project may have health and other effects but might not impose health care costs (e.g., nutrition, educational and environmental projects). Therefore, some assessment of how other types of benefits and costs should be traded against health and health care cost is required.

A project which has effects on health and health care costs but also imposes costs on private consumption (Δ*c*_*ct*_), or offers private consumption benefits (when Δ*c*_*ct*_ < 0), is illustrated in Table 2a. The time stream of health benefits net of the health opportunity losses in column 5 combines the health benefits and additional health care costs of the project. The net effect on consumption (in column 6) is the consumption costs (column 4) net of the impact on consumption of the health opportunity losses associated with the additional health care costs of the project. Therefore, once other effects beyond health and health care costs are included, some assessment of the consumption opportunity costs of the additional health care costs (*k*_*ct*_) is also required^10^. The net effects of the project can then be reported as two time streams of net health and net consumption effects (columns 5 and 6).^11^

**Table 2a:** Reporting the effects of a project on health, health care costs and consumption

Effects of the project				Effects on health	Effects on consumption
(1) Time	(2) Additional health benefits	(3) Additional health care costs	(4) Consumption costs	(5) Net health benefits	(6) Net consumption costs
1	Δ*h*_1_	Δ*c*_*h*__1_	Δ*c*_*c*__1_	Δ*h*_1_ – Δ*c*_*h*__1_*/k_h_*_1_	Δ*c*_*c*__1_ + *k*_*c*__1_ · Δ*c*_*h*__1_
—	—	—	—	—	—
*t*	Δ*h*_*t*_	Δ*c*_*ht*_	Δ*c*_*ct*_	Δ*ht* – Δ*c*_*ht*_/*k*_*ht*_	Δ*c*_*ct*_ + *kct* · Δ*c*_*ht*_
—	—	—	—	—	—
*T*	Δ*h*_*T*_	Δ*c*_*hT*_	Δ*c*_*cT*_	Δ*h*_*T*_ – Δ*c*_*hT*_/*k*_*hT*_	Δ*c*_*cT*_ + *k*_*cT*_ · Δ*c*_*hT*_

**Table 2b:** Expressing the net effects of a project as consumption, health and health care costs

Net effects			
(1) Time	(2) Equivalent consumption effects	(3) Equivalent health effects	(4) Equivalent health care resources
1	*V*_*h*__1_ (Δ*h*_1_ – Δ*ch*_1_/*k*_*h*__1_) – (Δ*c*_*c*__1_ + *k*_*c*__1_ · Δ*c*_*h*__1_)	(Δ*h*_1_ – Δ*c*_*h*__1_*/k_h_*_1_) – (Δ*c*_*c*__1_ + *k*_*c*__1_ · Δ*c*_*h*__1_)*/V_h_*_1_	*k*_*h*__1_ ((Δ*h*_1_ – Δ*c*_*h*__1_*/k_h_*_1_) – (Δ*c*_*c*__1_ + *k*_*c*__1_ · Δ*c*_*h*__1_)*/V_h_*_1_)
—	—	—	—
*t*	*V*_*ht*_(Δ*ht* – Δ*c*_*ht*_/*k*_*ht*_) – (Δ*c*_*ct*_ + *k*_*ct*_ · Δ*c*_*ht*_)	(Δ*h*_*t*_ – Δ*c*_*ht*_/*k*_*ht*_) – (Δ*c*_*ct*_ + *k*_*ct*_ · Δ*c*_*ht*_)/*V*_*ht*_	*k*_*ht*_((Δ*h*_*t*_ – Δ*c*_*ht*_/*k*_*ht*_) – (Δ*c*_*ct*_ + *k*_*ct*_ · Δ*c*_*ht*_)/*V*_*ht*_)
—	—	—	—
*T*	*V*_*ht*_(Δ*h*_*T*_ – Δ*c*_*hT*_/*k*_*hT*_) – (Δ*c*_*cT*_+ *k*_*cT*_ · Δ*c*_*hT*_)	(Δ*h*_*T*_ – Δ*c*_*hT*_/*k*_*hT*_) – (Δ*c*_*cT*_+ *k*_*cT*_ · Δ*c*_*hT*_)/*V*_*hT*_	*k*_*hT*_((Δ*h*_*T*_ – Δ*c*_*hT*_/*k*_*hT*_) – *(*Δ*c*_*cT*_+ *k*_*cT*_ · Δ*c*_*hT*_)/*V*_*hT*_)

Adopting an explicit consumption value of health (*υ*_*ht*_) allows the quantification and conversion of multiple effects to a common numeraire while reflecting the opportunity costs of constraints on health care expenditure. This is illustrated in Table 2b where the net health and consumption effects of the project (columns 5 and 6 of Table 2a) can be expressed as time streams of equivalent health effects (column 3), discounted at *r*_*s*_ – *gk*_*h*_; equivalent health care resources (column 4), discounted at *r*_*s*_; or the equivalent net consumption effects (column 2), discounted at *r*_*c*_.^12^

### 2.2 The objective of the project is to improve welfare

Traditionally the economic evaluation of social projects (e.g., Boadway & Bruce, 1984) adopts a view of social welfare resting on individual preferences revealed through markets or modified by an explicit welfare function. BCA is often founded on this more traditional approach and regards the purpose of any type of project, including those that require health care resources, as improving a broader notion of welfare rather than health or other explicitly stated social objectives. This type of analysis tends to be less well represented in the evaluation of health and medical care projects, partly due to the difficulty of decision-making bodies being willing to identify a welfare function carrying some broad consensus, particularly if health is felt to be unlike other goods (e.g., Broome, 1978; Sen, 1979; Brouwer et al., 2008; Arrow, 2012). Nevertheless, health must inevitably be traded with other welfare arguments, most notably consumption, by social planners whilst taking account of the constraints on health and other public expenditure they face.

BCA reports time streams of benefits and costs as their equivalent consumption values which represent the amount of consumption required to compensate for the costs of the project and the additional consumption that would be required to forego the benefits offered. The results of this type of analysis can be reported as the benefit-cost ratio or net present value of the project. If consumption and health are the only arguments or are separable from others (Gravelle et al., 2007) then the time stream of equivalent net consumption effects of the project illustrated in Table 2b (see column 2) or the equivalent consumption value of the benefits and costs of the project illustrated in Table 1 (see columns 8 and 9) are the estimates required for a BCA of these projects.

Conducting analysis in this extensive way (illustrated in Tables 1, 2a and 2b) ensures that any changes in the consumption value of health benefits and losses (*V*_*ht*_) are already explicitly included in how the time streams of effects have been valued. Similarly, any expected changes in the health and other opportunity costs of health care expenditure (*k*_*ht*_, *k*_*ct*_) have already been explicitly accounted for in the time stream of health and consumption effects (see column 2 of Table 2b). Once all the health and consumption effects of the project are expressed as equivalent time streams of consumption they can be discounted at a social time preference rate for consumption (*r*_c_). The sum of the discounted time stream of the equivalent net consumption effects is the net present value of the project.

An alternative to this more extensive approach would be to try and account for changes in the consumption value of health and the opportunity costs of health care expenditure through discounting. For example, for the project illustrated in Table 1, the discount rate for Δ*h*_*t*_ could be amended to reflect expected growth in the consumption value of health, *gV*_*h*_, by reducing the discount rate applied to health benefits (*r*_*c*_ – *gV*_*h*_). The discount rate applied to Δ*c*_*ht*_ would also need to be amended to reflect both growth in the consumption value of health opportunity losses and any expected growth in a “threshold” that reflects the health opportunity costs of heath expenditure (*r*_*c*_ – *gV*_*h*_ + *gk*_*h*_) (Claxton et al., 2011).^13^ This differential or dual discounting implicitly accounts for changes in the value of health and changes in the marginal productivity of health expenditure as well as time preference. This and other forms of dual discounting create potential for confusion and become more difficult when changes in the consumption opportunity costs of health care expenditure must be accounted for and when these parameters do not grow at a constant rate. The separate and explicit accounting for each of these effects illustrated in Table 2b would appear more transparent, accountable and comparable.^14^

How to think about time preference for equivalent consumption effects is well established and well worked through the Ramsey Rule (*r*_*c*_ = *δ* + *ηg*_*c*_). This includes pure time preference (*δ*, i.e., time preference for utility) and a wealth effect (*ηg*_*c*_) which reflects the relative weight attached to consumption in future compared to the current period. Individual choices do reveal forms of pure time preference, however, there are good, albeit disputed, reasons to set revealed pure time preference aside when making social choices that will have effects on current and future populations (Stern, 2008; Nordhaus, 2007; Arrow et al., 2013). The wealth effect in the Ramsey Rule requires some assessment of the growth in future consumption (*g*_*c*_) and the weight that ought to be attached to it (*η*). This can be cast in a number of ways (e.g., based on individual diminishing marginal utility of consumption) and appeal to different forms of evidence (Groom & Maddison, 2018). However, when considering social choices about projects which have impacts on current and future populations, it might be best thought of as a form of inequality aversion where expectations of future growth in consumption means that additional consumption for future beneficiaries should be given less weight than the same additional consumption for current beneficiaries. The important thing to note is that *r*_*c*_ will always be country specific because even if *η* is common (and it need not be) *r*_*c*_ will be determined by expectations about future consumption growth which are likely to differ.

### 2.3 What is the distinction between CEA and BCA?

The explicit assessment of the relative value of other effects shows that the distinction between a CEA which accounts for wider effects and a BCA which incorporates the opportunity of cost or shadow prices of existing constraints is more apparent than real. Both require the same assessment of the same key parameters in Tables 2a and 2b. Although much of the applied work to inform decision-making bodies has adopted a narrower health care system perspective (in part due to a concern for the perceived cost of conflicts with other important social objectives that are more difficult to fully specify and quantify), a broader ‘societal’ or multisectoral perspective in CEA is possible and is required and recommended by a number of decision-making bodies.^15^

What distinguishes BCA and CEA is a choice of whether social values ought to reflect those implied by the outcome of legitimate processes (e.g., government setting budgets for health care) or a notion of welfare founded on individual preferences or an explicit welfare function. For example, the former suggests a social time preference rate for health of *r*_*s*_ – *gk*_*h*_ and the latter, *r*_*c*_ – *gV*_*h*_. The distinction is whether the social value is expressed by *k*_*t*_ or *V*_*t*_ and whether it is the opportunity cost of financing health care or the welfare arguments that underpin the Ramsey Rule that justify discounting.^16^

The purpose is not to recommend which choice ought to be made but to clearly set out the implications for the common parameters that need to be assessed. The implication for discounting policy, whether conducting BCA or CEA, is that it becomes even more difficult and opaque to try and embed all these relevant arguments in how health, health care and other costs are discounted. The quantification and conversion of the time streams of multiple effects to a common numeraire (illustrated in Table 2b) avoids embedding multiple arguments in the discount rate for health and health care costs. For example, when it is believed to be important to explicitly quantify other impacts beyond measures of health and public health expenditure it would be appropriate to convert all effects into time streams of their equivalent consumption gains and losses, while reflecting the opportunity costs of existing constraints. These time streams of consumption benefits and costs can then be discounted at a social time preference rate for consumption based on the Ramsey Rule. The separate and explicit accounting for these arguments allows clarity about the parameters that need to be assessed, available evidence to be identified and used transparently and consistently, while preserving the possibility of accountable deliberation about evidence, values and unquantified arguments in decision-making processes.

## 3 Evidence available to inform key parameters and possible default estimates

The common conceptual framework for CEA and BCA set out in Section 2 identifies the key parameters that need to be assessed to express the time streams of effects in a common numeraire of equivalent consumption gains and losses, which can then be discounted at the social time preference rate for consumption, *r*_*c*_. Each of these parameters, including *r*_*c*_, are likely to be country specific. As a consequence, the net present value of a project which has effects across a number of countries will be the sum of the country specific net present values (see Table 4).

### 3.1 Health opportunity costs of health care expenditure (kht)

The problem of estimating a cost-effectiveness “threshold” that represents expected health opportunity costs is the same as estimating the relationship between changes in health care expenditure and health outcome.^17^. Estimates of the marginal productivity of health expenditure in producing health (QALYs) are becoming available for some high-income countries based on approaches to estimation which exploit within country data (Martin et al., 2008; Claxton et al., 2015a; Vallejo-Torres et al., 2016; Edney et al., 2018). The proportionate effect on all-cause mortality of proportionate changes in health expenditure (outcome elasticities) has also been estimated in higher income countries (Vallejo-Torres et al., 2016; Edney et al., 2018) using similar approaches to estimation. Others have used a different approach to identification which exploits exogenous elements in how funds are allocated within the UK National Health Service and have reported similar estimates (Andrews et al., 2017; Claxton et al., 2018). This evidence from high-income settings can be used to give some indication of possible values in lower income countries (Woods et al., 2016) based on a number of assumptions about income elasticity of demand for health and the relative “under funding” of health care systems. This type of extrapolation suggests that cost per QALY is likely to be less than gross domestic product (GDP) per capita in middle-income countries and substantially lower in low-income countries.

The effect of different levels of health care expenditure on mortality outcomes has been investigated in a number of published studies using country level data, many including LMICs (Gallet & Doucouliagos, 2017). The challenge is to control for all the other reasons why mortality might differ between countries in order to isolate the causal effect of differences in health expenditure. This is a particular challenge even if available measures are complete, accurate and unbiased because health outcomes are likely to be influenced by expenditure (increases in expenditure improve outcomes), but outcomes are also likely to influence expenditure (e.g., poor outcomes may prompt greater efforts and increased expenditure) (Nakamura et al., 2016). This problem of endogeneity, as well as the inevitable aggregation bias, risks underestimating the health effects of changes in expenditure.

Instrumental variables have been used in a number of studies to try and overcome this problem and estimate outcome elasticities for all-cause adult and child mortality, by gender, as well as survival, disability and DALYs (e.g., Bokhari et al., 2007). These estimated elasticities have been used to provide country specific cost per DALY averted values for 123 countries, taking account of measures of a country’s infrastructure, donor funding, population distribution, mortality rates, conditional life expectancies (all by age and gender), estimates of disability burden of disease and total health care expenditure (Ochalek et al., 2015). These estimates have recently been updated (Ochalek et al., 2018) and work is underway to assess how cost per DALY averted is likely to evolve with changes in health care expenditure and consumption growth.

There are considerable estimation challenges even in high-income settings where good quality data are more readily available. A particular challenge is to find suitable and valid instruments to overcome the problem of endogeneity. This, combined with the risk of aggregation bias and incomplete data, makes estimates based on country level data particularly uncertain.

#### 

##### Possible default estimates (*k*_*ht*_)

Some initial quantitative judgement about the likely health opportunity costs and how they may evolve is unavoidable whether conducting CEA or BCA. These judgements could be informed by current initial estimates in LMICs. However, the framework of analysis in Ochalek et al. (2018) can be applied to the results of any study thought to identify a more plausible effect on mortality of changes in health expenditure, whether they are based on country level or within country data. Any initial country specific default estimates can be refined and updated as other estimates emerge, ideally using within country data where this is possible.

### 3.2 Consumption opportunity costs of health care expenditure (*k*_*ct*_)

The consumption opportunity costs associated with health care expenditure require either direct evidence of the impact of changes on health care expenditure on net production (i.e., the value of additional production net of additional consumption) or estimates of the impact that changes in health are likely to have on net production in the rest of the economy (which with evidence from 3.1 the former can be derived from the latter).

Attempts to estimate and explicitly account for these nonhealth opportunity costs of health expenditure are particularly limited, even in high-income settings, but do exist.^18^ There are no explicit estimates for other countries, but a wide literature already exists at a microlevel (e.g., health and labour market outcomes) and at a macrolevel (e.g., health and economic growth) that could be marshalled to derive estimates of the likely productive effects of changes in health relevant to different settings. These types of estimates could provide some default assessment of the net production effects likely to be associated with the particular type of health benefits offered by a project. Importantly, they can also be linked to evidence of health opportunity costs in 3.1 to estimate the consumption opportunity costs of health care expenditure.

#### 

##### Possible default estimates (*k*_*ct*_)

In the absence of marshalling existing but disparate evidence, a default assumption of 1 (1 dollar spent on health care delivers 1 dollar in net production or consumption opportunities) might be a reasonable, albeit conservative, assumption in LMIC settings given the very limited evidence currently available. Although there is little evidence about how this aspect of opportunity costs is likely to evolve, a default assumption that the real value of the net production effects of the health effects of changes in health expenditure will grow at the same rate as consumption may not be unreasonable.

### 3.3 Consumption value of health and its evolution over time (*V*_*ht*_)

There is a large literature which has used stated preferences (contingent valuation and discrete choice experiments) to estimate the consumption value or willingness to pay for a measured improvement in health (QALYs gained) (e.g., Pinto-Prades et al., 2009; Mason et al., 2009). Recent reviews of this literature reveal wide variation in values (Vallejo-Torres et al., 2016; Ryen & Svensson, 2015; Thokala et al., 2018). These estimates reflect the demand for health, rather than a “supply side” assessment of health opportunity costs. Most estimate how much consumption an individual is willing to give up to improve their own health. A few try to elicit how much individuals believe society should pay to improve health more generally. A wider literature, that extends beyond health, estimates the value per statistical life (VSL) based on how much consumption individuals are willing to give up to reduce their mortality risk (Hammitt, 2000; Robinson et al., 2016, 2019). Some studies are based on stated preferences but others identify situations where individuals make choices that imply a value, e.g., revealed preferences in the labour market. There are relatively few studies of willingness to pay for nonfatal risk reductions (Robinson & Hammitt, 2018). Nonetheless, a cost per DALY can be derived from VSL studies based on the age and gender distribution, conditional life expectancies and quality of life norms. However, this requires strong assumptions that are unlikely to reflect individual preferences (Robinson & Hammitt, 2018).^19^

Most of this literature reports values relevant to high-income countries. However, some patterns that emerge are also likely to be relevant to LMICs: estimates based on VSL studies tend to be higher than those based on willingness to pay for a QALY gained; values are not proportional to the scale of health gains and differ depending on whether health gains are through quality improvement or survival benefits (Robinson & Hammitt, 2018; Robinson et al., 2019).^20^.

Although there is limited direct empirical evidence which provides values in lower income settings, there is some evidence about how values differ with income, which might be used to extrapolate from high to low-income settings (Hammitt, 2017; Robinson et al., 2019). However, adjusting for income is only one of a number of factors that are likely to influence values (Hammitt, 2017). Reviews of the literature that have investigated the relationship between the VSL and income (e.g., Hammitt & Robinson, 2011; Viscusi & Masterman, 2017; Masterman & Viscusi, 2018; Robinson et al., 2019) suggest that the consumption value of health increases with income and that an income elasticity of 1 would not be unreasonable (Masterman & Viscusi, 2018). However, the income elasticity for fatal and nonfatal risk reductions is likely to differ (Robinson & Hammitt, 2018; Robinson et al., 2019). Therefore, income elasticities from VLS studies may not fully reflect how the consumption value of a QALY gained or DALY averted changes with income.

#### 

##### Possible default estimates (*V*_*ht*_)

A simple but reasonable assessment of how *V*_*ht*_ is likely to evolve could be based on growth in consumption (which is already required and embedded in the wealth effect of the Ramsey Rule) and assumptions about the income elasticity of demand for health. An income elasticity of demand for health of 1 might be a reasonable, albeit conservative, default assumption in which case *V*_*ht*_ would grow at the same rate as consumption. This could be compared with a less conservative scenario based upon an income elasticity of 1.5. Other scenarios could be justified based on evidence that income elasticity is likely to differ in particular settings.

### 3.4 Other constrained sectors (*V*_*xt*_/*k*_*xt*_)

Health expenditure is not the only category of public expenditure which is constrained. Therefore, the effects of a project on other types of public expenditure ought to reflect their opportunity costs in the same way as health expenditure. One way to do this is to use the evidence that is available for the health sector to shadow price other forms of public expenditure. Estimates of the consumption value of health tend to be higher than available estimates of a “supply side” assessment of health opportunity costs (Vallejo-Torres et al., 2016). This suggests a common discrepancy between the demand and supply side of health care systems. For example, if these estimates are regarded as an appropriate expression of social value, the difference between *V*_*ht*_ and *k*_*ht*_ would indicate that health care from collectively pooled resources is ‘underfunded’ compared to individual preferences about health and consumption.^21^ It is consistent with the view that the public funding of health care is not matching individual preferences and public expectations of their health care system. However, given the difficulties faced in the public financing of health care, and the welfare losses associated with socially acceptable means of taxation, this is what might be expected and especially so in lower income settings where the difficulties of public financing are more acute.^22^ The balance of evidence suggests that *V*_*ht*_/*k*_*ht*_ > 1. This ratio is the shadow price for public health expenditure, i.e.,the value of one public health dollar relative to one private consumption dollar.^23^ Therefore, estimates of *V*_*ht*_ and *k*_*ht*_, which are already required, could also be used to shadow price other forms of public expenditure.

#### 

##### Possible default estimates (*V*_*xt*_/*k*_*xt*_)

Estimates of *V*_*ht*_/*k*_*ht*_ in the health sector might be used to shadow price other forms of public expenditure (where the equivalent estimates for that sector are absent) since resource allocation and expenditure decisions by government and other ministries would be expected to equalize this ratio across sectors (*x*) given an overall constraint on total public expenditure, i.e., it may not be unreasonable to assume *V*_*ht*_/*k*_*ht*_ = *V*_*xt*_/*k*_*xt*_ when considering impacts on public sector *x*.

### 3.5 Time preference for consumption (*r*_*c*_)

A social time preference rate for consumption based on the Ramsey Rule (*r*_*c*_ = *δ* + *ηg*_*c*_) includes pure time preference (*δ*) and a wealth effect (*ηg*_*c*_) which was discussed in Section 2.2. Pure time preference rates of 0–1% could be regarded as reasonable (Drupp et al., 2018), however, whether or not a revealed pure time preference should be set aside when considering projects with intergenerational effects turns on disputed ethical questions.^24^ The wealth effect in the Ramsey Rule reflects an assessment of the expected growth in consumption (*g*_*c*_) and the weight that ought to be attached to consumption in the future compared to the current period (*η*).

There is some revealed preference evidence to inform *η* in high-income countries (Groom & Maddison, 2018), and some empirical evidence drawn from a survey of expert opinion on long term decision-making (Drupp et al., 2018). However, the balance of this evidence in high-income settings suggests that there is some element of inequality aversion with values of 1 < *η* < 2 not being unreasonable sGroom & Maddison, 2018). There are also possibilities of obtaining revealed values for *η* through other social choices (e.g., the progressivity of tax and benefit systems) (Evans & Sezer, 2002). Such concerns for intraas well as intertemporal equity could lead to a focus on inequality adjusted growth such as using the growth rate of the median household rather than the mean per capita growth rate to estimate *r*_*c*_ (Emmerling et al., 2017). Little direct evidence of *η* exists for LMICs; nonetheless country specific default estimates of *r*_*c*_ are possible because even if *η* is common (and it need not be) it will be determined by expectations about economic growth which are likely to differ between countries with different expectations about economic growth.

#### 

##### Possible default estimates

A conservative default assumption to establish country specific estimates of *r*_*c*_ would be to apply *η* = 1 to available estimates of *g*_*c*_ (reported as expected growth in measures of national income per capita for that country), i.e., the discount rate applied to time streams of equivalent consumption effects is *g*_*c*_. This could be compared with a less conservative scenario based on *η* = 2, where *r*_*c*_ would be twice the expected growth in consumption. Other scenarios could be justified based on evidence or reasoning of why *η* is likely to differ in specific contexts or based on different judgements about the prospects of future economic growth by social planners. As evidence for values of *η* specific to LMICs evolves and estimates of economic growth are revised these defaults can be updated. This can also be compared to a wealth effect based on expected growth in median income where those are available.

### 3.6 Catastrophic, macroeconomic and project specific risk

#### 

##### Catastrophic risk

Truly catastrophic risk is best thought of as the probability of an event that represents total catastrophe for the whole of society so those who were to receive the welfare effects of a public policy or programme no longer exist. When cast in this way it excludes events which could be described as ‘catastrophic’ but where some recovery might be possible even if this requires assistance from others (other countries, global bodies). This is important, as although a ‘catastrophic’ event where recovery is possible may have a major impact on growth and whether the payoffs from particular projects are realized, these impacts are unlikely to be the same for all projects. Therefore, these types of ‘catastrophic’ but recoverable risks are best included in how macroeconomic risk and project specific risks are accounted for rather than being embedded in a common adjustment to the discount rate for all projects.

There are sources for probabilities of truly catastrophic events where recovery would not be possible (Chapman, 2004; Stern, 2007). The probabilities are relatively small and if included would add little to a common discount rate for consumption effects. Given the other more influential sources of uncertainty in specifying reasonable default values for a common discount rate for consumption affects it might be reasonable to set aside truly catastrophic risks. If catastrophic risk is included, it should be based on an estimate of the probabilities of truly catastrophic events where recovery would not be possible (⩽0.1%).

##### Macroeconomic risk

When all effects of a project are expressed as streams of consumption benefits and costs then discounting using the social time preference rate for consumption (*r*_*c*_) would be appropriate. When project net benefits are uncorrelated with the macroeconomy (aggregate consumption growth), some decline in *r*_*c*_ over longer time horizons will be needed due to persistent uncertainty in the consumption growth element of the wealth effect of the Ramsey Rule (Arrow et al., 2013; Cropper et al., 2014; Freeman & Groom, 2016; Freeman et al., 2015). In essence the declining term structure of discount rates reflects a societal demand for precautionary saving and is an expression of prudence: saving for a rainy day induced by macroeconomic downturns. The impact on net present values is modest over shorter terms but is more significant when there is greater uncertainty about economic growth. A similar effect on *r*_*c*_ occurs when there is a risk of low probability but significant shocks (Barro, 2009; Gollier, 2014).

The time horizons for the evaluation of many projects with health effects are often less than 30 years or generally do not extend much beyond that. For example, insofar as a project impacts mortality risk, the time horizon for costs and benefits may only extend to the survival of the cohort of current beneficiaries. However, projects that change the dynamics of infectious disease and/or require commitment of irrecoverable costs also require an assessment over the survival of future incident cohorts that will be affected or will benefit from the investment.

Therefore, the use of *r*_*c*_ without adjustment for macroeconomic risk may be a reasonable default assumption for projects with time horizons less than 30 years. Where there are longer time horizons, or where macroeconomic risk is greater and increases more rapidly with term (as is more likely in LMICs), declining rates might be required but should be based on uncertainty in consumption growth rather than behavioural evidence of time preference. Since growth and uncertainty about that growth will be country specific, any decline in *r*_*c*_ will also be country specific.

##### Project specific risk

Considerable efforts have been made in the evaluation of health projects to characterize all sources of uncertainty, value the consequences and establish how these should inform project choice, e.g., whether the approval should be delayed until further research is conducted or until sources of uncertainty resolve over time. The impact of irrecoverable costs and the real option value of delay have been examined, as well as the impact of approval on the opportunities to acquire evidence that would benefit future patient populations (Drummond et al., 2015, Chapter 11). This type of analysis starts to unpack the reasons for the appearance of risk aversion in project choice.

Some project evaluations may have already accounted for the consequences of some project specific risks in ways that others may not. In any event, these risks and their consequences necessarily differ by project so should certainly not be embedded in a common discount rate for the consumption effects. Therefore, where possible, project specific risks should be included in how the expected time streams of consumption equivalent effects of the project are estimated. If these risks have been accounted for in this way it could be argued that they can be discounted at a risk free rate (Arrow & Lind, 1970) and no project specific risk premium would be required. This would be reasonable if government or other funders of the project can diversify risk (e.g., across many taxpayers) and that project specific risks are uncorrelated with those in the wider economy. If they are correlated then a project specific risk premium may be required to account for the interaction between project specific and macroeconomic risk (Baumstark & Gollier, 2014).

This interaction is not often considered in the evaluation of social projects but is a well-established feature of how capital assets are priced. The parallel for social projects is to consider the relationship between the uncertainty in the project’s payoffs and uncertainty in future macroeconomic conditions. For example, a project with payoffs that are countercyclical (provides greater than expected payoffs when growth and consumption is lower than expected) is more valuable than a project with the same expected payoffs, but which offers procyclical risks (offers poorer than expected outcomes when growth is lower than expected).

This can be reflected in a project specific risk premium (beta) which depends on the correlation between project payoffs and realized consumption growth, i.e., the project specific discount rate will be higher than *r*_*c*_ when the correlation is positive (beta > 0) and lower than *r*_*c*_ when it is negative (beta < 0), whether or not *r*_*c*_ has been adjusted for macroeconomic risk (Gollier, 2012). This also has implications for how the discount rate changes with term due to macroeconomic risk. For example, although rates will decline for a risk free project (beta = 0), they may increase with term for projects that are strongly procyclical (beta > 0) and increase more quickly with greater uncertainty in economic growth. The magnitude of these effects also depends on how any correlation tends to be concentrated. For example, if it is concentrated in low probability but high impact events, such as a severe fall in consumption, the risk premium will be highly positive or negative, which can significantly reduce or increase the value of the project (Barro, 2009).

This may be especially important in LMIC settings, where uncertainty about economic growth and the possibility of low probability but high impact events may be higher than in high-income countries. Uncertainty about the outcomes of projects in LMICs might also be greater and more strongly correlated with macroeconomic risk. Therefore, an important question is whether a project is likely to be pro- or countercyclical and whether any correlation is concentrated in low probability high impact events. For example, the present value of a project which is likely to offer greater payoffs in times of war, economic crisis or severe epidemic will be substantially higher than indicated by discounting at *r*_*c*_. In the absence of established estimates of betas for health projects and a lack of experience in the field in estimating them, a qualitative indication of whether or not projects are likely to be strongly pro- or countercyclical and how these cyclical effects are likely to be concentrated would be a useful starting point for deliberation by decision makers, while further research is conducted on how the effects of these interactions might be best quantified for these types of project relevant to LMICs.

## 4 Recommendations, reporting and further research

### 4.1 Summary of possible estimates and default assumptions

The possible estimates and default assumptions for the parameters identified in Section 2 and discussed in Section 3, are summarized in Table 3.

**Table 3:** Key parameters and possible estimates and default assumptions

Key parameters		Possible estimates and default assumptions
Health opportunity costs of health care expenditure in each period (*t*)	*k*_*ht*_	Estimates reported in Ochalek et al. (2018) could provide initial default estimates of cost per DALY averted Projections of these estimates based on estimates of health expenditure and consumption growth are possible These initial estimates can be refined and updated as other country specific estimates emerge
Consumption opportunity costs of health care expenditure in each period (*t*)	*k*_*ct*_	A conservative default assumption of 1 (1 dollar spent on health care delivers 1 dollar in net production) A conservative default assumption that the real value of the net production effects will grow at the same rate as consumption
Consumption value of health in each period (*t*)	*υ*_*ht*_	Country specific estimates of *V*_*ht*_, see Robinson et al. (2019) and Robinson and Hammitt (2018) Growth in *V*_*ht*_ can be based on growth in consumption (*gc*) and the income elasticity of demand for health A conservative scenario using an income elasticity of demand for health of 1 (*V*_*ht*_ will grow at *gc*) Scenario using an income elasticity of demand of 1.5
Opportunity costs for other sectors (*x*) in each period (*t*) Social rate of time preference for consumption	*υ*_*xt*_/*k*_*xt*_	Default assumption that *v*_*ht*_/*k*_*ht*_ = *υ*_*xt*_/*k*_*xt*_ when considering impacts on other public sectors
	*rc*	A normative assumption of zero pure time preference for social choices is not unreasonable (*δ* = 0) Two scenarios based on alternative assumptions of inequality aversion can be used A conservative scenario based on *η* = 1 (*rc* = *gc*) Alternative scenario based on *η* = 2 (*rc* = 2*gc*) Other scenarios can be based on evidence of why *η* is likely to differ in specific contexts or different judgements about *gc*
Catastrophic risk		If catastrophic risk is included it should be based an estimate of the probability of truly catastrophic events where recovery would not be possible (≤0.1%)
Macroeconomic risk		Use of *rc* without adjustment for macroeconomic risk may be a reasonable for projects with shorter time horizons (⩽30 years) For longer time horizons or where macroeconomic risk is greater, declining rates may be required but should be based on uncertainty in consumption growth
Project specific risk		Where possible project specific risks should be included in how the time streams of consumption equivalent effects of the project are estimated A qualitative indication of whether projects are likely to be strongly pro or counter cyclical should be provided Further research is required on how the interaction of project specific and macroeconomic risk might be best quantified for the types of project relevant to LMICs

These parameters, which are common to CEA and BCA, depend, directly or indirectly, on expectations about the growth in consumption. For example, *r*_*c*_ and growth in the consumption value of health both depend directly on *g*_*c*_. The change in health and other opportunity costs associated with health care expenditure will also, in part, be determined by expectations about economic growth. The relative value of impacts on different dimensions of health outcome (morbidity and mortality) is also likely to be country specific and will change with economic growth. This has two implications. First, since *g*_*c*_ will be country specific, all these parameters will also be country specific, including *r*_*c*_, which has important implications for aggregating effects of a project that is relevant to a number of different jurisdictions (see Section 4.2). Second, it is important than any assessment of *g*_*c*_ is consistently applied to inform all the key parameters that depend on it, so that any change in these expectations, or any alternative judgements about *g*_*c*_, feeds through into all the relevant parameters and is used consistently throughout.

### 4.2 Reporting and aggregating effects

Extensive reporting is recommended, as illustrated in Tables 1, 2a and 2b. Reporting the results of CEA and BCA in this way makes explicit the assessments required. This enables the impact of alternative, but plausible, assumptions to be explored and analysis to be updated as better estimates evolve. The quantification and conversion of the time streams of effects into their equivalent health, health care cost or consumption effects avoids embedding multiple arguments in discounting policies. The separate and explicit accounting for these arguments provides clarity about the parameters that need to be assessed, available evidence to be identified and used transparently and consistently, while preserving the possibility of accountable deliberation about evidence, values and unquantified arguments in decision-making processes.

Some projects and supranational investments will have effects across different counties where all these key parameters will differ, e.g., when global bodies make recommendations, purchase health technologies, or prioritize the development of new ones. Other projects and national investments will have effects across jurisdictions (e.g., states or provinces) within a particular country where only some of these parameters differ (e.g., *k*_*ht*_). Therefore, the net present value of a project which has effects across a number of countries will be the sum of the country specific net present values rather than the sum of effects across countries discounted at some common rate. This is illustrated in Table 4 for a project with effects on health, health care costs and consumption in three countries or jurisdictions (A, B and C). The country specific effects must be transformed into country specific time streams of equivalent consumption and then discounted at the country specific rate for consumption. The country specific Net Present Values (NPV) can then be summed to indicate the global NPV of a project with effects in a number of countries.

**Table 4:** Reporting the effects of a project with impacts on more than one jurisdiction

Equivalent consumption effects across countries or jurisdictions			
	Country A	Country B	Country C
Net present value	$\sum_{t=1}^{T}\frac{\upsilon_{h,t}^{A}\left[\text{Δ}h_{t}^{A}-\frac{\text{Δ}c_{h,t}^{A}}{k_{h,t}^{A}}\right]-\left[\text{Δ}c_{c,t}^{A}+k_{c,t}^{A}\cdot \text{Δ}c_{c,t}^{A}\right]}{{\left(1+r_{c}^{A}\right)}^{t}}$	$\sum_{t=1}^{T}\frac{\upsilon_{h,t}^{B}\left[\text{Δ}h_{t}^{B}-\frac{\text{Δ}c_{h,t}^{B}}{k_{h,t}^{B}}\right]-\left[\text{Δ}c_{c,t}^{B}+k_{c,t}^{B}\cdot \text{Δ}c_{c,t}^{B}\right]}{{\left(1+r_{c}^{B}\right)}^{t}}$	$\sum_{t=1}^{T}\frac{\upsilon_{h,t}^{C}\left[\text{Δ}h_{t}^{C}-\frac{\text{Δ}c_{h,t}^{C}}{k_{h,t}^{C}}\right]-\left[\text{Δ}c_{c,t}^{C}+k_{c,t}^{C}\cdot \text{Δ}c_{c,t}^{C}\right]}{{\left(1+r_{c}^{C}\right)}^{t}}$
Global net present value	$\sum_{t=1}^{T}\frac{\upsilon_{h,t}^{A}\left[\text{Δ}h_{t}^{A}-\frac{\text{Δ}c_{h,t}^{A}}{k_{h,t}^{A}}\right]-\left[\text{Δ}c_{c,t}^{A}+k_{c,t}^{A}\cdot \text{Δ}c_{c,t}^{A}\right]}{{\left(1+r_{c}^{A}\right)}^{t}}+\sum_{t=1}^{T}\frac{\upsilon_{h,t}^{B}\left[\text{Δ}h_{t}^{B}-\frac{\text{Δ}c_{h,t}^{B}}{k_{h,t}^{B}}\right]-\left[\text{Δ}c_{c,t}^{B}+k_{c,t}^{B}\cdot \text{Δ}c_{c,t}^{B}\right]}{{\left(1+r_{c}^{B}\right)}^{t}}+\sum_{t=1}^{T}\frac{\upsilon_{h,t}^{C}\left[\text{Δ}h_{t}^{C}-\frac{\text{Δ}c_{h,t}^{C}}{k_{h,t}^{C}}\right]-\left[\text{Δ}c_{c,t}^{C}+k_{c,t}^{C}\cdot \text{Δ}c_{c,t}^{C}\right]}{{\left(1+r_{c}^{C}\right)}^{t}}$		

### 4.3 Suggestions for further research

An initial quantitative assessment of health opportunity costs and how they are likely to evolve is possible based on the balance of evidence from studies using country level data. Projections of these estimates can be linked to expectations of consumption growth which determines the social time preference rate and growth in the consumption value of health. More recent estimates of the health effects of changes in health care expenditure suggest that larger effects tend to be identified when using within country data and especially at a disease area level. Therefore, extending the estimation of outcome elasticities using within country data to more health care systems would be particularly valuable. Attempts to estimate the consumption opportunity cost of health expenditure are particularly limited. Nonetheless, a wide literature already exists at a microlevel (e.g., health and labour market outcomes) and at a macrolevel (e.g., health and economic growth) which could be marshalled to derive estimates of the likely productive effects of changes in health relevant to different settings.

Estimates of country specific expected growth in consumption are available and can inform a number of parameters, including *r*_*c*_. However, measures of inequality adjusted growth such as the difference in the growth of median and mean income are not readily available for LMICs. There is little direct evidence to inform the weight that might be attached to future compared to current consumption (*η*) for LMICs. There are, however, possibilities of obtaining revealed values for *η* through other social choices (e.g., the progressivity of tax and benefit systems). The evidence to support estimates of the consumption value of health in particular LMICs and income elasticities of demand for health are considered in more detail in Robinson and Hammitt (2018) and Robinson et al. (2019).

The use of *r*_*c*_ without adjustment for increasing uncertainty about future consumption may be a reasonable default assumption for projects with shorter time horizons. However, a review of estimates of *g*_*c*_ relevant to LMICs could identify the circumstances where declining rates should be applied. This could be used to develop tools which would provide appropriate declining rates for *r*_*c*_ based on *g*_*c*_, measures of uncertainty in *g*_*c*_ by term, *η* and an initial assumption of beta = 0.

Whether projects are likely to be pro-, or countercyclical is not often considered in the evaluation of social projects and there is a lack of experience of estimating this type of risk premium (betas) for the types of projects considered in LMICs. Therefore, a first step would be to illustrate, with case studies, how a qualitative indication of whether or not projects are likely to be strongly pro-, or countercyclical might be made and how these correlations are likely to be concentrated. This might also identify the characteristics of projects and circumstances where quantifying the interaction of project and macroeconomic risk is likely to be particularly important in project choice and how betas might be estimated for these types of project.

## Footnotes

1 See Drummond et al. (2015, Section 2.4.3, pp. 33–38).

2 This is commonly illustrated by a comparison of terminal and present values. The cost per quality adjusted life year (QALY) of a project with immediate costs and additional health benefits all occurring at a future point in time is the same whether costs are expressed at their terminal value when the health benefits occur, or discounting the health benefits back to their present value at the same rate (Nord, 2011).

3 See Drummond et al. (2015) page 108–112.

4 See Drummond et al. (2015), Section 2.4.1, pp. 27–31 and Section 4.2.1, pp. 79–83.

5 This is equivalent to asking whether the cost per QALY the policy offers is less than the cost effectiveness “threshold” (Δ*c*_*h*_/Δ*h < k_h_*), so long as the “threshold” used to judge cost-effectiveness reflects the likely health opportunity costs.

6 This is the time preference for health, as distinct from pure time preference (for utility) or social rate of time preference (SRTP) for consumption.

7 This approximation is based on the plausible assumption that *r*_*h*_ and *g*_*k*_ are small.

8 There are reasons to set aside explicit and quantitative consideration of other effects if they are likely to conflict with other important social arguments that are difficult to specify, let alone quantify, e.g., equity and the benefits of social solidarity offered by collectively funded health care. This is the explicit decision that has been taken in the UK by the National Institute for Health and Care Excellence after considering the benefits and potential costs of quantifying these wider effects in the decision-making process (Claxton et al., 2015*b*).

9 The welfare losses associated with socially acceptable forms of taxation means it costs more than one private dollar to raise one public dollar. This marginal cost of public funds means it is unlikely that public health expenditure would be increased to the point where *k*_*ht*_ = *V*_*ht*_· *V*_*h*_/*k*_*h*_ indicates the value of public health expenditure relative to private consumption, or the shadow price of public health expenditure (see Section 3.4), which, if greater than an assessment of the marginal cost of public funds (e.g., 1.3 to 1.5), would suggest that health expenditure should be increased.

10 If there are consumption effects of changes in health there will also be consumption opportunity costs due to the health opportunity losses associated with health care costs of the project. If these consumption effects run only through the health effects of health expenditure, then *kc* will reflect the consumption effects of changes in health. Insofar as health expenditure has a positive impact on economic growth compared to other forms of public expenditure then restricting attention to the consumption effects of changes in health may underestimate the consumption opportunity costs of health care expenditure (see Section 3.2).

11 It should be noted that attempts to estimate and explicitly account for the consumption opportunity costs of health care expenditure are particularly limited, even in high-income settings, but do exist (Claxton et al., 2015*b*). Although there is currently little evidence in lower income setting to support such assessment some default assumptions based on what is already known about the relationship between changes in health and economic growth should be possible.

12 Converting the time stream health care costs into a time stream of health effects using *k*_*ht*_ before valuing them in consumption using *V*_*ht*_ and discounting at *rc* has strong parallels with the shadow price of capital approach in BCA, where the time stream of any displaced private investment is first converted into a time stream of consumption losses, which, along with the other direct consumption effects of the project, are discounted at *rc*.

13 This approximation is based on the plausible assumption that *r*_*h*_, *g*_*υ*_ and *g*_*k*_ are small.

14 The UK Department of Health recommends that health opportunity costs are dealt with explicitly and separately from discounting. Nonetheless they recommend a discount rate of 1.5% for health and health care costs and 3.5% for other effects, which embeds the expectation that the consumption value of health will grow at 2%. This happens to nullify the wealth effect in the UK Treasury social time preference rate based on the Ramsey Rule.

15 See Drummond et al. (2015), Section 4.5.3, pp. 112–116. For example National institute for Health and care Excellence requires a primary analysis from the perspective of the health care system. However, an analysis that includes other effects can be considered and is required for public health interventions and programmes. Other decision-making bodies in the Netherlands and Sweden require a broader perspective to be adopted as the primary analysis. A societal perspective was recommended as reference case analysis by the Washington Panel (Gold et al., 1996), alongside a health care system perspective. The recent update to this guidance (Neumann et al., 2016) recommends analysis from both a societal and health care system perspective.

16 The actual differences may be modest if *g*_*k*_ and *g*_*υ*_ are similar and the real rate at which government can borrow is regarded as a reasonable proxy for STPR as some argue it is (Council of Economic Advisers, 2017).

17 See Drummond et al. (2015), Section 4.3, pp. 83–94; Section 4.3.3.1, pp. 95–95.

18 For example, as part of efforts to inform value based pricing of branded medicines the UK Department of Health undertook work to estimate the “wider social benefits” associated with changes in health outcome which could be linked to evidence of health opportunity costs to estimate the net production opportunity costs of changes in health expenditure. The evidence in the UK suggests that a marginal pound in the NHS budget provides 92p worth of net production gains (see Appendix B of Claxton et al., 2015*b*).

19 It should be noted that there are good reasons to suppose that *V*_*h*_ · Δ*h* will not fully reflect individual welfare when *Vh* is some population average and Δ*h* has not measured all dimensions of outcome that are of value to the individual and/or where multiple dimensions have been combined imposing assumptions that do not reflect individual preferences (e.g., QALY and DALY). As a consequence measures of willingness to pay for a unit change in a measure of health may not reflect an individual’s willingness to pay for the prospect of effects across many dimensions of outcome, some of which may not have been measured. Similarly VSL studies estimate the willingness to pay for a change in a particular type of risk which is likely to differ by the level of risk faced the scale of the change in risk. Finally, individual willingness to pay will differ across individuals, so adopting a particular average value for the population (e.g., at average income) is unlikely to reflect the welfare effects for this population. Nonetheless, estimates of the health effects of projects (e.g., mortality, survival, morbidity, DALY, QALY) are more likely to be available than individual willingness to pay for the prospect of effects the project offers. Similarly the opportunity costs of health expenditure are more easily approximated by *V*_*h*_ (Δ*c*_*h*_/*k*_*h*_) than willingness to pay for the prospect of effects of a change in health expenditure might offer. For these reasons estimates of the willingness to pay for measured health effects are useful, even though all aspects of individual welfare may not be fully reflected in *V*_*h*_ · Δ**_*h*_.

20 A problem of insensitivity to scale in stated preference studies is not restricted to health but can occur in the valuation of other attributes such as environmental quality.

21 For example, the UK DH has adopted *£*15,000 per QALY to assess health opportunity costs and until recently *£*60,000 per QALY as an estimate of the consumption value of health based on deriving QALY effects from VSL estimates. This would suggest that one health care pound is worth 4 pounds of private consumption effects, which is especially important when there are other impacts which fall outside constrained public expenditure.

22 The welfare losses associated with socially acceptable ways to raise public finance means there is a marginal cost of public funds, i.e., it costs more than one private dollar to raise one public dollar. Estimates of the marginal cost of public funds of 1.3 to 1.5 are not unreasonable (e.g., Ruggeri, 1999; Dahlby, 2008) and suggest it is unlikely that public health expenditure should be increased to the point where *k*_*ht*_ = *V*_*hts*_.

23 This shadow price will be higher if improvements in health also have positive consumption effects because the value of additional public health expenditure includes the health effects of expenditure (*k*_*h*_) valued at *V*_*h*_, plus the consumption effects of health expenditure (*kc*).

24 For example, if it is regarded as unethical to treat people differently only because they happen to appear at different points in time then pure time preference could be set aside on the grounds of impartial utilitarianism.
